# Predicting gestational age using neonatal metabolic markers

**DOI:** 10.1016/j.ajog.2015.11.028

**Published:** 2016-04

**Authors:** Kelli K. Ryckman, Stanton L. Berberich, John M. Dagle

**Affiliations:** a Department of Epidemiology, University of Iowa, Iowa City, IA; b Department of Pediatrics, University of Iowa, Iowa City, IA; c State Hygienic Laboratory at the University of Iowa, Coralville, IA

**Keywords:** fetal growth, neonatal metabolism, preterm birth

## Abstract

**Background:**

Accurate gestational age estimation is extremely important for clinical care decisions of the newborn as well as for perinatal health research. Although prenatal ultrasound dating is one of the most accurate methods for estimating gestational age, it is not feasible in all settings. Identifying novel and accurate methods for gestational age estimation at birth is important, particularly for surveillance of preterm birth rates in areas without routine ultrasound dating.

**Objective:**

We hypothesized that metabolic and endocrine markers captured by routine newborn screening could improve gestational age estimation in the absence of prenatal ultrasound technology.

**Study Design:**

This is a retrospective analysis of 230,013 newborn metabolic screening records collected by the Iowa Newborn Screening Program between 2004 and 2009. The data were randomly split into a model-building dataset (n = 153,342) and a model-testing dataset (n = 76,671). We performed multiple linear regression modeling with gestational age, in weeks, as the outcome measure. We examined 44 metabolites, including biomarkers of amino acid and fatty acid metabolism, thyroid-stimulating hormone, and 17-hydroxyprogesterone. The coefficient of determination (R^2^) and the root-mean-square error were used to evaluate models in the model-building dataset that were then tested in the model-testing dataset.

**Results:**

The newborn metabolic regression model consisted of 88 parameters, including the intercept, 37 metabolite measures, 29 squared metabolite measures, and 21 cubed metabolite measures. This model explained 52.8% of the variation in gestational age in the model-testing dataset. Gestational age was predicted within 1 week for 78% of the individuals and within 2 weeks of gestation for 95% of the individuals. This model yielded an area under the curve of 0.899 (95% confidence interval 0.895−0.903) in differentiating those born preterm (<37 weeks) from those born term (≥37 weeks). In the subset of infants born small-for-gestational age, the average difference between gestational ages predicted by the newborn metabolic model and the recorded gestational age was 1.5 weeks. In contrast, the average difference between gestational ages predicted by the model including only newborn weight and the recorded gestational age was 1.9 weeks. The estimated prevalence of preterm birth <37 weeks’ gestation in the subset of infants that were small for gestational age was 18.79% when the model including only newborn weight was used, over twice that of the actual prevalence of 9.20%. The newborn metabolic model underestimated the preterm birth prevalence at 6.94% but was closer to the prevalence based on the recorded gestational age than the model including only newborn weight.

**Conclusions:**

The newborn metabolic profile, as derived from routine newborn screening markers, is an accurate method for estimating gestational age. In small-for-gestational age neonates, the newborn metabolic model predicts gestational age to a better degree than newborn weight alone. Newborn metabolic screening is a potentially effective method for population surveillance of preterm birth in the absence of prenatal ultrasound measurements or newborn weight.

Accurate estimation of gestational age is important for perinatal care and research. Clinically, predicting gestational age during pregnancy is important for determining the treatment and management of pregnancies that may end in preterm birth (<37 weeks’ completed gestation). Preterm birth is the leading cause of child death, ahead of infectious disease, worldwide with the greatest rates in low-resource regions such as West Africa.^1^ Population estimates of gestational age are extremely important for determining the burden of preterm birth and small-for-gestational age, particularly in low-resource settings.^1^ Accurate gestational age estimates are necessary for identifying the causes and risk factors for preterm birth and small-for-gestational age as well as evaluating interventions that may be used to prevent these conditions in the future. Identifying areas with greater-than-average preterm and small-for-gestational age rates can aid health professionals in targeting interventions where they would have the largest impact.

There are several methods commonly used for estimating gestational age during pregnancy. One such method, ultrasound dating, is based on estimating gestational age by measuring the size of the fetus in early pregnancy. Another commonly used method, particularly in areas without access to ultrasound technologies, is estimating gestational length based on a woman’s last menstrual period. Last menstrual period often is inferior to ultrasound dating, because it relies on a woman remembering the date of her last menstrual cycle.^2^, ^3^, ^4^ Although ultrasound dating is becoming increasingly common in the United States, it is not currently practical in most developing regions of the world or for women receiving little or no prenatal care. Gestational age dating by fetal ultrasound also is not robust for neonates who are small or large for their gestational age.^5^, ^6^

For underdeveloped areas in which women do not have access to prenatal care, gestational age can be estimated after birth. Dubowitz or Ballard examinations estimate gestational age by the use of standardized scoring systems based on physical and neuromuscular characteristics of the newborn infant.^7^, ^8^ Gestational age estimates based on either the Dubowitz or Ballard criteria are less precise than obstetric estimates.^9^, ^10^ In fact, neonatal-derived gestational age estimates most often overestimate the number of infants born less than 40 weeks’ gestation while underestimating the number of infants born at or greater than 40 weeks’ gestation.^9^, ^10^ Birthweight also can be used to estimate gestational age but is limited in the same way as neonatal examinations and is not robust in small- or large-for-gestational age infants.^11^

Gestational age is correlated strongly with many developmental and metabolic processes and is a strong predictor of neonatal outcome.^12^ Several studies have demonstrated altered maternal and cord blood amino acid and fatty acid metabolites in pregnancies that end in preterm birth or the birth of a low birthweight or small for gestational age neonate.^13^, ^14^, ^15^ In addition, there are distinct urinary metabolic patterns in preterm neonates compared with their term counterparts.^16^ We and others have shown that metabolites related to amino acid and fatty acid metabolism measured 24−72 hours after birth are vastly different among very preterm (<32 weeks), moderately preterm (32−36), and term (≥37 weeks) neonates.^17^, ^18^ Newborn metabolic screening via the use of tandem mass spectrometry has long been recognized as a critical public health initiative to identify mostly treatable but individually rare inborn errors of metabolism.^19^ Newborn metabolic screening captures data from a variety of biomarkers, including amino acids, free carnitine, and acylcarnitines. We hypothesized that metabolic and endocrine markers captured by routine neonatal screening could improve gestational age estimation in the absence of prenatal ultrasound technology. This technique would have practical application for surveillance of preterm birth at a population level when prenatal care is limited.

## Materials and Methods

### Study population

We performed a retrospective analysis of 238,315 newborn metabolic screening records collected by the Iowa Newborn Screening Program between 2004 and 2009. Forty-four metabolites were measured on all subjects during the entire study period, including 2 enzymes (biotinidase and galactose-1-phosphate uridyl transferase), 2 hormones (thyroid-stimulating hormone [TSH] and 17-hydroxyprogesterone [17-OHP]), 9 amino acids, 30 acylcarnitines, and free carnitine (C0) (Supplementary Table 1). Blood spot specimens were collected, dried, and handled as part of routine clinical care according to the Clinical Laboratory Standards Institute guidelines.^20^ At the time of neonatal screening, the health care provider records the gestational age in weeks, the sex of the infant, current weight in grams, if the infant is currently on total parenteral nutrition, and age of the newborn in hours. This information is included with each newborn screening specimen. Data such as delivery mode or maternal characteristics were not available. The method of gestational dating, ie, last menstrual period or fetal ultrasound, is provider dependent and is not distinguished on the newborn screen record. All specimens were analyzed as part of the Iowa Newborn Screening Program by the State Hygienic Laboratory in Ankeny, Iowa. Screening procedures in Iowa are based on previously established methodology.^18^, ^19^

The State Hygienic Laboratory identified multiple gestations by examining birth date, gestational age, mother’s first name, and facility identification number. The data were deidentified by the State Hygienic Laboratory and provided for use in this study. Approval for use of the deidentified data was obtained from the Iowa Department of Public Health and a waiver of consent from the Institutional Review Board at the University of Iowa (IRB#200908793).

Only initial newborn screening specimens, not repeats, were included in the analysis. We excluded screening records with missing gestational age data (n = 5749) or those with a recorded gestational age day outside the range of 20−45 completed weeks (n = 108). Records for specimens that were rejected by the screening laboratory as being of poor quality (n = 2445) were excluded from analysis. The remaining dataset consisted of 230,013 neonatal metabolic screening records. To determine the final performance of the predictive model, the data were randomly split into a model-building dataset (n = 153,342) and a model-testing dataset (n = 76,671). The predictive model was created using the model-building dataset and the performance of this model was then evaluated in the model-testing dataset.

### Statistical analysis

Univariate analysis was performed with each metabolite and gestational age. Linearity between gestational age and single metabolite levels was inspected visually by plots of the residuals vs the predicted values. To address nonlinearity between each metabolite and gestational age, squared terms and then the cubed terms were included for each model. We performed multiple linear regression modeling with gestational age, in weeks, as the outcome measure, using metabolites that were significant in the univariate analysis. The regression was estimated by the use of ordinary least squares. In the model-building dataset, all metabolites significant at *P* < .01 from the univariate models were included in the initial model, and significant terms (*P* < .05) were retained for subsequent modeling. Squared and cubed terms of significant metabolites were included successively in the model after which nonsignificant (*P* > .05) terms were removed. Cubic terms were examined only when squared terms were significant.

Next, within the model-building dataset, we determined whether the final selected model was robust in the presence of covariates that could affect the prediction of gestational age by the metabolic panel. These covariates included the child’s sex, age at time of sample collection (in hours, month, and year of sample collection), neonatal weight at time of screening in grams, weight for gestational age categorized as small-for-gestational age (<10th percentile for each gestational age week), large-for-gestational age (>90th percentile for each gestational age week), and average-for-gestational age and multiple gestation. Residuals vs the predicted values were inspected visually for the relationship between gestational age and age at time of screening as well as gestational age with weight. To address nonlinearity between age and weight with gestational age, squared terms and then the cubed terms were included for each model.

We performed a sensitivity analysis excluding newborns identified as potentially affected with an endocrine disorder or inborn error of metabolism, ie, one or more metabolite levels exceeded the threshold considered within the normal range for a healthy newborn. Statistical outliers also were evaluated with studentized residuals by excluding those observations with residuals less than −1.96 and greater than 1.96. The coefficient of determination (R^2^) and the root-mean-square error (RMSE) are presented for each regression model. All analyses were performed in STATA version 12.0 (College Station, TX).

The final model was used to predict gestational age in the model-testing dataset (n = 76,671). The coefficient of determination (R^2^) and the RMSE are presented for the entire model-testing dataset as well as stratified by sex and weight for gestational age. Sensitivity and specificity were calculated by gestational age cut-points (in weeks) for prediction of preterm birth less than <37 weeks’ gestation compared with term birth (≥37 weeks). Because birthweight can also predict gestational age, we evaluated the sensitivity and specificity with each preterm birth outcome in a model that included only infant weight and its squared and cubed terms. Next, we evaluated the sensitivity and specificity of each preterm birth outcome using a model that contained the final set of metabolites plus weight and their subsequent squared and cubed terms to examine the extent to which adding weight improves prediction over that based on the metabolites alone.

## Results

### General characteristics of the population and the newborn metabolic model

Gestational age distributions were comparable in both the model-building and model-testing datasets, with 8.9% of the total population being born preterm (<37 weeks) (Supplementary Table 2). Distributions of neonatal weight, small- and large-for-gestational age, sex, total parenteral nutrition, and age at time of screening were similar in both the model-building and model-testing datasets (Supplementary Table 2). All univariate analyses between each metabolite (including the squared and cubed terms) with gestational age were significant at *P* < .01 (data not shown). Within the model-building dataset, the full model including 1 categorical variable (biotinidase), 43 continuous metabolite measurements, and their squared and cubed terms produced comparable performance statistics (R^2^ = 53.2%, RMSE = 1.3) to the metabolic model including only significant terms (R^2^ = 53.1%, RMSE = 1.3). Therefore, our final newborn metabolic linear regression model consisted of 88 parameters, including the intercept, 37 metabolite measures, 29 squared metabolite measures, and 21 cubed metabolite measures (Table 1). Metabolites in the final model included TSH, 17-OHP, galactose-1-phosphate uridyl transferase, 7 amino acids, and 27 acylcarnitines.

### Model performance in model-building dataset

The newborn metabolic model explained 53.1% of the variation in gestational age in the model-building dataset. The average difference between gestational ages predicted by the newborn metabolic model and the recorded gestational age was 1.3 weeks (Table 2). The prediction of gestational age by the use of the newborn metabolic model was robust to inclusion of covariates. Specifically, including month and year of sample collection, multiple gestations, infant age at time of sample collection in hours, infant sex, and weight for gestational age did not improve the prediction of gestational age. Each of these covariates alone, explained very little of the variation in gestational age (R^2^ < 5%; RMSE ∼ 1.9 weeks). Sensitivity analyses demonstrate that gestational age estimated by the newborn metabolic model was not impacted by exclusion of newborns with potential endocrine disorders or inborn errors of metabolism, newborns receiving total parenteral nutrition or multiple gestations (Table 2). The removal of statistical outliers unsurprisingly improved model performance (R^2^ = 55.4; RMSE = 1.1); however, there was no reason to suspect that the metabolite values of these individuals were not true values and we chose not to remove these individuals from further analyses.

### Comparison of the newborn metabolic model to including only neonatal weight in the model-building dataset

In the model-building dataset, neonatal weight alone explained 54.5% of the variation in gestational age. The average difference between gestational ages predicted by neonatal weight alone and the recorded gestational age was 1.3 weeks (Table 2). Including neonatal weight in the newborn metabolic model improved the difference between the predicted gestational ages and recorded gestational age by 0.2 weeks and explained 66.1% of the variation in gestational age. Inclusion of neonatal metabolite measurements explained an additional 12% of the variation in gestational age above and beyond neonatal weight (Table 2).

### Performance of the newborn metabolic model in the model-testing dataset

In the model-testing dataset, the newborn metabolic model estimated gestational age within 1 week for 78% of the individuals and within 2 weeks of gestation for 95% of the individuals. One individual had a predicted gestational age of 172 weeks, which was likely attributable to the fact that this individual had multiple abnormal metabolite levels. We excluded this individual from subsequent evaluation of model performance. In the model-testing dataset, the newborn metabolic model (n = 76,670) predicted 52.8% of the variation in gestational age. The average difference between the gestational ages predicted by the newborn metabolic model and the recorded gestational age was 1.3 weeks, which was comparable (R^2^ = 52.8%, RMSE = 1.3) to the model including the excluded individual (n = 76,671).

### Comparison of the newborn metabolic model to including only neonatal weight in the model-testing dataset

In the model-testing dataset neonatal weight alone explained 54.6% of the variation in gestational age. The average difference between gestational ages predicted by neonatal weight alone and the recorded gestational age was 1.3 weeks. Similar to the findings in the model-building dataset, including neonatal weight in the newborn metabolic model improved the difference between the predicted gestational ages and recorded gestational age by 0.2 weeks in the model-testing dataset. The model including neonatal weight with the newborn metabolic model explained 66.2% of the variation in gestational age in the model-testing dataset. Therefore, inclusion of neonatal metabolite measurements explained an additional 12% of the variation in gestational age above and beyond neonatal weight.

The overall estimated prevalence of preterm birth (5.0%) when we used the newborn metabolic model was slightly closer to the percentage of preterm birth (8.9%) estimated with the recorded gestational age than the model including only neonatal weight (4.7%). The newborn metabolic model yielded an area under the curve (AUC) of 0.899 (95% confidence interval [95% CI] 0.895−0.903) in differentiating those born preterm (<37 weeks) from those born term (≥37 weeks) (Figure and Supplementary Table 3). The newborn metabolic model offers slight improvement in identifying those born preterm (<37 weeks) over the model including only neonatal weight (AUC 0.881, 95% CI 0.876−0.886). Including neonatal weight in the newborn metabolic model provides the best estimation of gestational age with an AUC of 0.938 (95% CI 0.934−0.941).

### Performance of the newborn metabolic model in neonates born very preterm or small-for-gestational age

The newborn metabolic model underestimated very preterm birth (<32 weeks) compared with the model including only neonatal weight (Table 3). There was no increased ability to discriminate between very preterm birth (<32 weeks) and all other births (≥32 weeks) using the newborn metabolic model over the model including only neonatal weight (Table 3 and Supplementary Figure).

The most evident difference in model performance was in small- and large-for-gestational age neonates. The average difference between gestational ages predicted by the newborn metabolic model and the reported gestational age was 1.5 weeks compared with the model with only neonatal weight (RMSE = 1.9 weeks). In large-for-gestational age neonates, the average difference between gestational ages predicted by the newborn metabolic model and the reported gestational age was 1.4 weeks, an improvement over the model with only neonatal weight (RMSE = 1.8 weeks).

Improvement over the model including only neonatal weight also was observed when we examined the predicted prevalence of preterm birth in the subset of small-for-gestational age neonates (Table 4). The model including only neonatal weight overestimated the prevalence of very preterm birth (<32 weeks) by over twice that of the actual prevalence (3.45% vs 1.61%, respectively), whereas the newborn metabolic model only marginally underestimated the prevalence of very preterm birth compared with the actual prevalence (1.00% vs 1.61%, respectively). For preterm birth <37 weeks’ gestation, the model including only neonatal weight estimated the prevalence at 18.79% in small-for-gestational age neonates, over twice that of the actual prevalence of 9.20%. Again the newborn metabolic model underestimated preterm birth but was closer to the true prevalence at 6.94% than the models including only neonatal weight (Table 4).

## Comment

### Primary findings of the study

Our primary findings were as follows: (1) The newborn metabolic profile, as detected by routine newborn screening, can accurately predict gestational age to the same degree and slightly better than neonatal weight alone; (2) the newborn metabolic model predicts gestational age to a better degree than neonatal weight alone in small and large for gestational age neonates; (3) newborn screening measurements can be used for population surveillance of preterm birth rates in the absence of prenatal ultrasound measurements; and (4) models including both the metabolites and neonatal weight provide the best prediction of gestational age.

### Significance of gestational age estimation at birth

Medical care and treatment of preterm neonates is of critical public health importance. Globally, approximately 15 million babies are born preterm (<37 weeks’ gestation) and 1.1 million deaths are attributable to preterm birth.^11^, ^21^ Preterm birth remains one of the single greatest contributors to infant mortality in the United States^22^ and to disability-adjusted life years worldwide.^23^ Approximately, 60% of all preterm births occur in low-resource settings, including Africa and South Asia.^11^ Accurate gestational age estimation is not only important for clinical decisions regarding the management and treatment of specific pregnancies but also for identifying areas with particularly high rates of preterm birth. We present a novel method for estimating gestational age based on the metabolic profile of the newborn that offers improvement over prediction using neonatal weight alone.

### Newborn metabolic profiles, gestational age, and fetal growth

Previous studies by our group have identified TSH, 17-OHP, amino acids, and acylcarnitines as potentially important markers of gestational age.^18^ Other studies have shown similar results in maternal blood, cord blood, and neonatal urine.^13^, ^14^, ^15^, ^16^ Differences in these metabolites by gestational age often are attributed to fetal sickness, stress, and immaturity of kidney, adrenal gland, and liver function. Additionally, because of their unique anatomy (large surface to volume ratio and large head to body ratio) and physiology (hepatic immaturity), premature neonates often have disrupted glucose homeostasis and may require glucose infusion early in life to prevent hypoglycemia.^24^ A disruption in glucose homeostasis can lead to an increase in amino acids and acylcarnitines that are important intermediates in beta oxidation and the Kreb’s cycle such as the metabolites measured as part of newborn screening.

Neonatal metabolism, as represented by newborn screening measurements, is a function of not only gestational age but growth in utero*.*^25^, ^26^ This point is further supported by the ability of the metabolite markers to predict 37.3% of the variation in weight in the model-testing dataset. Additionally, the newborn metabolic model outperforms neonatal weight alone in predicting gestational age, particularly in small-for-gestational age neonates.

### Clinical significance of gestational age dating at birth

Neonatal metabolism is a function of not only in utero growth but also maternal metabolism reflected by the transfer of nutrients from the mother to the child via the placenta.^25^ Therefore, studies examining these specific biomarkers throughout pregnancy could provide insights into preterm birth etiology as well as better methods of gestational age dating, particularly in small or large for gestational fetuses. Determining gestational age postbirth is important for differentiating neonates who are preterm from those who are small-for-gestational age. The distinction between infants born preterm and average-for-gestational age, infants born preterm and small-for-gestational age, and term infants born small-for-gestational age is important because the morbidity and mortality risks differ between these groups.^27^, ^28^, ^29^, ^30^, ^31^ Metabolic profiling at birth is unlikely to provide immediate information for clinical decision making; however, for long-term follow-up, it may be important to distinguish accurately between the small-for-gestational age infant and the preterm, average-for-gestational age infant.

### Newborn metabolic profiles and accurate estimation of preterm birth rates

Reporting of gestational age on the US Standard Certificate of Live Birth has been based primarily on last menstrual period and often is used to report national rates of preterm birth.^32^ In 2014, however, the obstetric estimate will replace last menstrual period as the primary standard for reporting gestational age.^32^ The obstetric estimate is defined as the best estimate of the infant’s gestation in completed weeks and is based on all available information, including last menstrual period and fetal ultrasound measurements. Comparisons in reporting methods demonstrate a 2% reduction in the preterm birth rate when obstetric estimate is reported instead of last menstrual period.^32^ When the newborn metabolic model was used, our estimated preterm birth rate was about 3% less than the rate calculated based on the recorded gestational age, which was based on a mixture of gestational ages solely reported by last menstrual period and some reported by obstetric estimate. Further studies are needed that directly compare the metabolic model to the gestational age obtained by early ultrasound dating to accurately assess the validity of this method in estimating preterm birth.

### Newborn metabolic profiles and global surveillance of preterm birth

Global surveillance of preterm birth is important for improving intervention programs by targeting them to areas with the greatest rates of preterm birth. Our method of gestational age prediction based on newborn screening metabolites provides several important insights and implications for preterm birth surveillance. Estimating gestational age in low-resource areas is hampered by lack of prenatal care services and ultrasound dating.^11^ Measuring these metabolites from a dried blood spot specimen offers several advantages beyond gestational age dating alone. Newborn metabolic screening using tandem mass spectrometry has long been recognized as a critical public health initiative to identify often rare conditions that if detected early enough can be treated to avoid serious morbidity or death.^19^ Although in low-resource settings it is unlikely that the infrastructure could be easily established to ensure timely detection and treatment of these conditions, data are severely lacking on the incidence of these conditions in regions of the world including Sub-Saharan Africa.^33^ Understanding the incidence of these conditions around the world would aid in developing targeted screening tests that may be more readily incorporated in low-resource settings than tandem mass spectrometry.

### Strengths and limitations of our study

Our study was strengthened by the large sample size and the ability to validate our model in a subset of the data. Although our method generalizes to the population of Iowa, which is primarily white, validating in other populations, including low-resource countries such as Africa will be important. Our analysis of newborn screening records was retrospective and did not include evaluation of the birth certificate or medical record; therefore, we are limited to the gestational age and selected characteristics provided to the newborn screening program, such as weight, sex, and age at time of newborn screening. In addition, the gestational age was provided by the health care provider, and it was unknown whether this was based on last menstrual period, fetal ultrasound, or a combination of both. Previously, however, we have examined newborn screening data linked to medical record data for 523 preterm newborns^34^, ^35^ and found that gestational age by best obstetric estimate was highly correlated with the gestational age reported on the newborn screening card (rho=0.988) (data not shown). In fact, 98% of the records matched within 1 gestational age week to the best obstetric estimate (ie, prenatal ultrasound or last menstrual period in the absence of prenatal ultrasound) with 86% being an exact match. Similar concordance was true for weight at time of newborn screening, which was strongly correlated (rho=0.990) to birthweight. Approximately, 99% of newborn screening records reported weight within 500 g (∼1 pound) of the birthweight and 86% were within 100 g (∼0.2 pounds) of birthweight. In addition, we were limited to the metabolites provided on the newborn screening panel. There are additional measures such as glucose and T-cell receptor excision circle analysis that are currently being added to routine screening panels across the United States and are also shown to correlate strongly with gestational age.^36^

## Conclusion

Our novel method of estimating gestational age at birth using the newborn metabolic profile, derived from markers routinely captured by newborn screening, provides more information than neonatal weight alone, particularly in small-for-gestational age infants. Our method, however, still underestimates preterm birth prevalence, particularly very preterm birth. Therefore, it is important to examine the utility of our model with other methods of gestational age estimation at birth including Ballard and Dubowitz scoring systems as well as emerging technologies such as retinal and lens vessel scans. Establishing methods of gestational age dating at birth using metabolic profiling could also have substantial benefits for improving infant and child health that extend beyond the estimation of gestational age alone.

## Figures and Tables

**Supplementary Figure:**
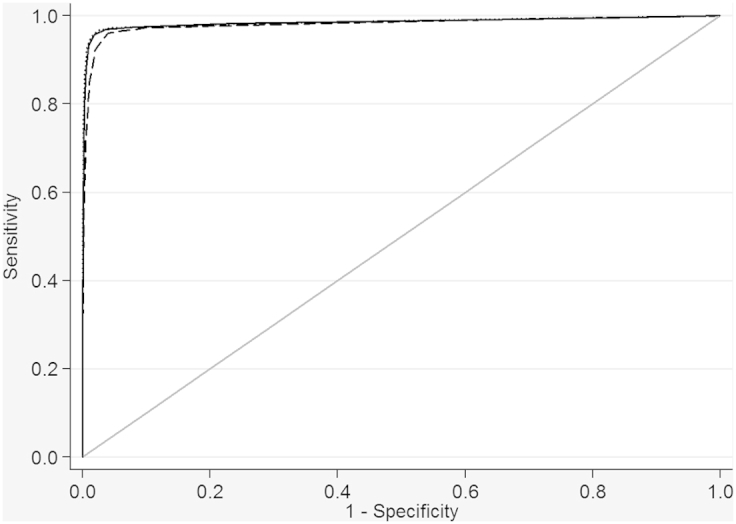
Receiver operator curves of metabolic and weight models for identifying those born preterm (<32 completed weeks) vs all others (≥32 completed weeks) Curves for weight at collection only model (*solid line*), metabolic final model (*dashed line*), and weight plus metabolic model (*dotted line*). *Ryckman. Metabolic predictors of gestational age. Am J Obstet Gynecol 2016*.

**Supplementary Table 1:** Enzymes and metabolites evaluated on every infant born between 2004 and 2009 by the Iowa Neonatal Screening Program

Enzymes and Hormones	Acylcarnitines (μmol/L)
17-Hydroxyprogesterone (17OHP - ng/mL)	Glutarylcarnitine (C5-DC)
Biotinidase (BIO – 3+, 2+ and 1+/0)	3-Hydroxyisovalerylcarnitine (C5-OH)
Galactose-1-Phosphate Uridyl Transferase (GALT - U/gHb)	Hexanoylcarnitine (C6)
Thyroid-stimulating hormone (TSH - mIU/L)	Methylglutarylcarnitine (C6-DC)
Amino acids (μmol/L)	Octanoylcarnitine (C8)
Alanine (ALA)	Octenoylcarnitine (C8:1)
Arginine (ARG)	Decanoylcarnitine (C10)
Citrulline (CIT)	Decenoylcarnitine (C10:1)
Glutamate (GLU)	Dodecanoylcarnitine (C12)
Isoleucine+Leucine (LEU)	Dodecenoylcarnitine (C12:1)
Methionine (MET)	Tetradecanoylcarnitine (C14)
Phenylalanine (PHE)	Tetradecenoylcarnitine (C14:1)
Tyrosine (TYR)	Tetradecadienoylcarnitine (C14:2)
Valine (VAL)	3-Hydroxytetradecanoylcarnitine (C14-OH)
Acylcarnitines (umol/L)	Palmitoylcarnitine (C16)
Carnitine free (C0)	Palmitoleylcarnitine (C16:1)
Acetylcarnitine (C2)	3-Hydroxypalmitoleylcarnitine (C16:1-OH)
Propionylcarnitine (C3)	3-Hydroxypalmitoylcarnitine (C16-OH)
Malonylcarnitine (C3-DC)	Stearoylcarnitine (C18)
Butyrylcarnitine+Isobutyrylcarnitine (C4)	Oleoylcarnitine (C18:1)
Methylmalonylcarnitine (C4-DC)	3-Hydroxyoleoylcarnitine (C18:1-OH)
Isovalerylcarnitine+Methylbutyrylcarnitine (C5)	Linoleoylcarnitine (C18:2)
Tiglylcarnitine (C5:1)	3-Hydroxystearoylcarnitine (C18-OH)

*Ryckman. Metabolic predictors of gestational age. Am J Obstet Gynecol 2016*.

**Supplementary Table 2:** Characteristics of Iowa Neonatal Metabolic Screening Data, 2004−2009

Variable	Model-building n = 153,342		Model-testing n = 76,671		Entire cohort n = 230,013	
n	%	n	%	n	%
Gestational age, completed wk						
20–31	1754	1.1	866	1.1	2620	1.1
32–36	12,218	8.0	5946	7.8	18,164	7.9
37–40	126,042	82.2	63,078	82.3	189,120	82.2
41–45	13,328	8.7	6781	8.8	20,109	8.7
Birth weight, g						
451–1499	1604	1.1	844	1.1	2448	1.1
1500–2499	10,036	6.5	4822	6.3	14,858	6.5
2500–3999	130,652	85.2	65,551	85.5	196,203	85.3
4000–5000	11,020	7.2	5437	7.1	16,457	7.2
Unknown	30	0.02	17	0.02	47	0.02
Weight for gestational age						
SGA	15,278	10.0	7502	9.8	22,780	9.9
AGA	122,477	79.9	61,412	80.1	183,889	80.0
LGA	15,253	10.0	7589	9.9	22,842	9.9
Unknown	334	0.2	168	0.2	502	0.2
Age at time of screening, h						
0–24	21,862	14.3	10,887	14.2	32,749	14.2
25–72	128,053	83.5	64,124	83.6	192,177	83.6
>72	3407	2.2	1649	2.2	5056	2.2
Unknown	20	0.01	11	0.01	31	0.01
Sex						
Male	78,121	51.1	39,230	51.2	117,351	51.0
Female	74,553	48.6	37,135	48.4	111,688	48.6
Unknown	668	0.4	306	0.4	974	0.4
Total parenteral nutrition						
Y	2049	1.3	1029	1.3	3078	1.3
N	151,293	98.7	75,642	98.7	226,935	98.7

*Ryckman. Metabolic predictors of gestational age. Am J Obstet Gynecol 2016*.

**Supplementary Table 3:** Sensitivity and specificity of metabolic models with and without weight for identifying those born preterm (<37 weeks) vs those born term (≥37 weeks) in the model-testing dataset

Preterm definition	Weight-only model AUC 0.8809 (95% CI 0.8762−0.8856)			Metabolic model AUC 0.8988 (95% CI 0.8947−0.9030)			Weight + metabolic model AUC 0.9376 (95% CI 0.9344−0.9407)		
Sensitivity	Specificity	Classified	Sensitivity	Specificity	Classified	Sensitivity	Specificity	Classified
<41	100.00%	0.00%	8.88%	99.96%	1.76%	10.49%	99.99%	0.88%	9.68%
<40	98.53%	19.49%	26.51%	98.56%	26.38%	32.79%	99.34%	28.17%	34.49%
<39	83.62%	81.05%	81.28%	89.94%	73.22%	74.71%	93.89%	77.74%	79.17%
<38	61.61%	95.81%	92.77%	66.87%	94.54%	92.09%	76.27%	95.27%	93.58%
<37	42.16%	98.97%	93.92%	45.24%	98.91%	94.15%	54.29%	99.12%	95.14%
<36	29.00%	99.70%	93.42%	30.92%	99.73%	93.61%	36.82%	99.80%	94.21%
<35	20.89%	99.89%	92.88%	21.81%	99.91%	92.97%	26.53%	99.91%	93.40%
<34	16.10%	99.92%	92.48%	14.74%	99.96%	92.39%	18.81%	99.95%	92.75%
<33	12.54%	99.94%	92.18%	9.70%	99.98%	91.96%	14.32%	99.97%	92.36%
<32	10.00%	99.95%	91.97%	6.59%	99.99%	91.69%	11.07%	99.98%	92.08%
<31	7.89%	99.97%	91.79%	4.42%	99.99%	91.50%	8.77%	99.99%	91.89%
<30	6.12%	99.98%	91.64%	2.97%	100.00%	91.38%	6.49%	100.00%	91.69%
<29	4.70%	99.98%	91.52%	1.79%	100.00%	91.27%	4.98%	100.00%	91.56%
<28	3.33%	99.99%	91.40%	1.03%	100.00%	91.20%	3.42%	100.00%	91.42%
<27	0.00%	100.00%	91.12%	0.00%	100.00%	91.12%	0.00%	100.00%	91.12%

*AUC*, area under the curve.

*Ryckman. Metabolic predictors of gestational age. Am J Obstet Gynecol 2016*.

**Figure:**
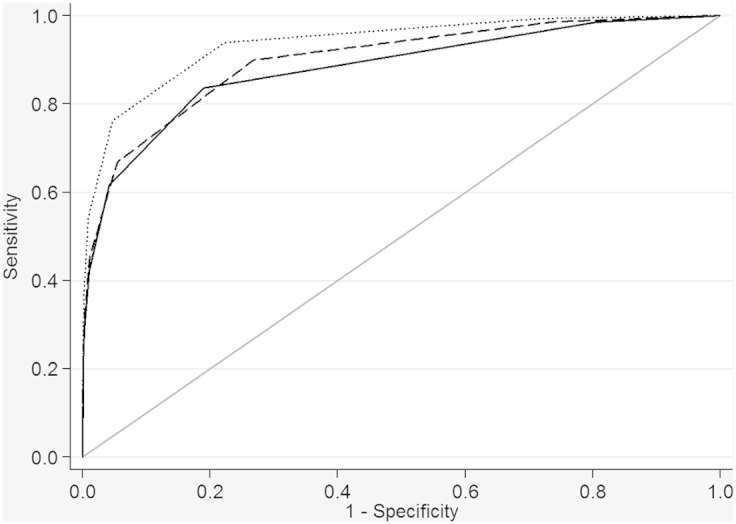
Receiver operator curves of metabolic and weight models for identifying those born preterm (<37 completed weeks) vs those born term (≥37 completed weeks) Curves for weight at collection only model (*solid line*), metabolic final model (*dashed line*), and weight plus metabolic model (*dotted line*). *Ryckman. Metabolic predictors of gestational age. Am J Obstet Gynecol 2016*.

**Table 1:** Final metabolite model in the model-building dataset (n = 153,342) for prediction of gestational age

Metabolite	Metabolite		Metabolite Squared		Metabolite Cubed	
Coefficient	SE	Coefficient	SE	Coefficient	SE
TSH	0.01	8.8×10^-4^	−8.8×10^-5^	1.2×10^-5^	1.3×10^-7^	2.3×10^-8^
17-OHP	−0.05	5.2×10^-4^	1.1×10^-4^	3.5×10^-6^	N/A	N/A
GALT	−0.07	0.01	3.5×10^-3^	7.5×10^-4^	N/A	N/A
ALA	9.6×10^-3^	2.7×10^-4^	−1.3×10^-5^	6.3×10^-7^	5.3×10^-9^	3.7×10^-10^
ARG	−0.02	1.5×10^-3^	2.9×10^-5^^a^	1.0×10^-5^	N/A	N/A
LEU	−0.01	3.3×10^-4^	1.7×10^-5^	8.3×10^-7^	−4.3×10^-9^	2.1×10^-10^
MET	0.03	1.2×10^-3^	−2.3×10^-4^	1.2×10^-5^	3.5×10^-7^	1.8×10^-8^
PHE	−0.01	8.3×10^-4^	4.2×10^-5^	4.7×10^-6^	−2.9×10^-8^	3.9×10^-9^
TYR	−5.3×10^-3^	1.2×10^-4^	N/A	N/A	N/A	N/A
VAL	0.02	6.0×10^-4^	−3.6×10^-5^	2.5×10^-6^	2.0×10^-8^	1.0×10^-9^
C2	−0.10	4.4×10^-3^	2.0×10^-3^	1.1×10^-4^	−1.2×10^-5^	8.5×10^-7^
C3	0.06	5.6×10^-3^	N/A	N/A	N/A	N/A
C4	−0.20	0.03	N/A	N/A	N/A	N/A
C5	−9.29	0.26	−6.93	0.78	6.81	0.43
C5:1	−3.89	0.56	N/A	N/A	N/A	N/A
C5-OH	−0.36	0.08	N/A	N/A	N/A	N/A
C3-DC	0.91	0.10	N/A	N/A	N/A	N/A
C4-DC	18.56	0.41	−50.43	1.67	40.19	1.92
C5-DC	−25.06	0.92	85.79	8.60	−118.09	23.90
C6	10.16	0.31	−2.24	0.42	N/A	N/A
C8	−1.47	0.20	0.11	0.02	−2.0×10^-3^	3.0×10^-4^
C8:1	13.29	0.50	−52.96	2.51	55.61	3.64
C10	4.99	0.32	−10.87	0.91	3.97	0.52
C10:1	−2.33	0.27	N/A	N/A	N/A	N/A
C12	2.47	0.19	−4.00	0.37	2.10	0.22
C12:1	5.17	0.30	−13.00	0.85	9.71	0.72
C6-DC	−4.20	0.36	9.65	2.13	N/A	N/A
C14	−3.03	0.32	1.94	0.48	N/A	N/A
C16	2.99	0.07	−0.58	0.02	0.04	1.5×10^-3^
C16:1	−9.73	0.55	18.06	1.63	−10.40	1.59
C18	−7.96	0.23	4.96	0.20	−1.02	0.05
C18:1	4.09	0.18	−1.70	0.11	0.24	0.02
C18:2	−4.22	0.19	3.01	0.35	−1.22	0.17
C14-OH	27.02	1.83	−287.34	35.03	N/A	N/A
C16-OH	10.91	2.58	−239.18	62.78	1585.56^a^	460.58
C16:1-OH	7.32	1.09	18.72^b^	8.87	N/A	N/A
C18:1-OH	3.84	0.82	N/A	N/A	N/A	N/A
Constant	36.72	0.11	N/A	N/A	N/A	N/A

*GALT*, galactose-1-phosphate uridyl transferase; *N/A*, not available; *17-OHP*, 17-hydroxyprogesterone *TSH*, thyroid-stimulating hormone.

All terms are significant at *P* < .001 unless otherwise noted.

*Ryckman. Metabolic predictors of gestational age. Am J Obstet Gynecol 2016*.

a *P*<.01

b .01≤*P*<.05.

**Table 2:** Percent of variation in gestational age explained by neonatal metabolic screening markers in the model-building dataset

Model description	No. model parameters^a^	No. observations	R^2^ (%)	RMSE
Final metabolite model	88	153,342	53.1	1.3
Final metabolite model excluding infants with abnormal neonatal screens	88	148,104	50.1	1.3
Final metabolite model excluding infants on total parenteral nutrition	88	151,293	47.9	1.3
Final metabolite model excluding statistical outliers as defined as those with residuals less than −1.96 and greater than 1.96	88	146,083	55.4	1.1
Final metabolite model + month and year of collection	104	153,342	53.4	1.3
Month and year of collection only	17	153,342	1.2	1.9
Final metabolite model + multiple gestation status (yes/no)	89	153,342	53.7	1.3
Multiple gestation status only	2	153,342	4.8	1.9
Final metabolite model including only singleton births	88	147,597	49.4	1.3
Final metabolite model + age at collection (hours)	91	153,322	53.1	1.3
Age at collection (hours) only	4	153,322	0.05	1.9
Final metabolite model in subset with nonmissing age at collection	88	153,322	53.1	1.3
Final metabolite model + infant sex	89	152,674	53.2	1.3
Infant sex only	2	152,674	0.02	1.9
Final metabolite model in subset with non-missing infant sex	88	152,674	53.1	1.3
Final metabolite model + infant weight, g	91	153,008	66.1	1.1
Infant weight, g only	4	153,008	54.5	1.3
Final metabolite model + weight for gestational age (AGA, LGA, and SGA)	90	153,008	53.1	1.3
Weight for gestational age (AGA, LGA, and SGA) only	3	153,008	0	1.9
Final metabolite model in subset with nonmissing weight	88	153,008	52.9	1.3

*AGA*, average-for-gestational age; *LGA*, large-for-gestational age; *RMSE*, root-mean-square error; *SGA*, small-for-gestational age.

*Ryckman. Metabolic predictors of gestational age. Am J Obstet Gynecol 2016*.

a Model parameters include the intercept term.

**Table 3:** Prevalence of gestational age groups in the model-testing dataset, n = 76,671

Gestational age	Actual gestational age			Metabolic model			Weight-only model		
n	%	Cum%	n	%	Cum%	n	%	Cum%
≤32	1177	1.54	0.00	674	0.88	0.00	891	1.16	0.00
33−34	1271	1.66	3.19	876	1.14	2.02	604	0.79	1.95
35−36	4364	5.69	8.88	2291	2.99	5.01	2089	2.72	4.67
37−38	18,927	24.69	33.57	20,994	27.38	32.39	15,306	19.96	24.64
39−40	44,151	57.59	89.62	50,600	66.00	97.51	57,613	75.14	98.62
>40	6781	8.84	100.00	1235	1.61	99.99	0	0.00	99.78
Missing	0.00	0.00	100.00	1	0.00	100.00	168	0.22	100.00

*Ryckman. Metabolic predictors of gestational age. Am J Obstet Gynecol 2016*.

**Table 4:** Prevalence of gestational age groups in the model-testing dataset for small-for-gestational age neonates, n = 7502

Gestational age	Actual gestational age			Metabolic model			Weight-only model		
n	%	Cum%	n	%	Cum%	n	%	Cum%
≤32	121	1.61	1.61	75	1.00	1.00	259	3.45	3.45
33−34	132	1.76	3.37	122	1.63	2.63	199	2.65	6.11
35−36	437	5.83	9.20	324	4.32	6.94	952	12.69	18.79
37−38	1831	24.41	33.60	2421	32.27	39.22	5861	78.13	96.92
39−40	4304	57.37	90.98	4481	59.73	98.95	231	3.08	100.00
>40	677	9.02	100.00	79	1.05	100.00	0	0.00	100.00

*Ryckman. Metabolic predictors of gestational age. Am J Obstet Gynecol 2016*.

## References

[1] Lawn J.E., Kinney M. (2014). Preterm birth: now the leading cause of child death worldwide. Sci Transl Med.

[2] Tunon K., Eik-Nes S.H., Grottum P. (1996). A comparison between ultrasound and a reliable last menstrual period as predictors of the day of delivery in 15,000 examinations. Ultrasound Obstet Gynecol.

[3] Kieler H., Axelsson O., Nilsson S., Waldenstrom U. (1993). Comparison of ultrasonic measurement of biparietal diameter and last menstrual period as a predictor of day of delivery in women with regular 28 day-cycles. Acta Obstet Gynecol Scand.

[4] Lynch C.D., Zhang J. (2007). The research implications of the selection of a gestational age estimation method. Paediatr Perinatal Epidemiol.

[5] Hadlock F.P., Deter R.L., Harrist R.B., Park S.K. (1984). Estimating fetal age: computer-assisted analysis of multiple fetal growth parameters. Radiology.

[6] Chervenak F.A., Skupski D.W., Romero R. (1998). How accurate is fetal biometry in the assessment of fetal age?. Am J Obstet Gynecol.

[7] Dubowitz L.M., Dubowitz V., Goldberg C. (1970). Clinical assessment of gestational age in the newborn infant. J Pediatr.

[8] Ballard J.L., Khoury J.C., Wedig K., Wang L., Eilers-Walsman B.L., Lipp R. (1991). New Ballard Score, expanded to include extremely premature infants. J Pediatr.

[9] Sanders M., Allen M., Alexander G.R. (1991). Gestational age assessment in preterm neonates weighing less than 1500 grams. Pediatrics.

[10] Alexander G.R., de Caunes F., Hulsey T.C., Tompkins M.E., Allen M. (1992). Validity of postnatal assessments of gestational age: a comparison of the method of Ballard et al. and early ultrasonography. Am J Obstet Gynecol.

[11] Blencowe H., Cousens S., Chou D. (2013). Born too soon: the global epidemiology of 15 million preterm births. Reprod Health.

[12] Blair E., Liu Y., Cosgrove P. (2004). Choosing the best estimate of gestational age from routinely collected population-based perinatal data. Paediatr Perinatal Epidemiol.

[13] Horgan R.P., Broadhurst D.I., Walsh S.K. (2011). Metabolic profiling uncovers a phenotypic signature of small for gestational age in early pregnancy. J Proteome Res.

[14] Heazell A.E., Bernatavicius G., Warrander L., Brown M.C., Dunn W.B. (2012). A metabolomic approach identifies differences in maternal serum in third trimester pregnancies that end in poor perinatal outcome. Reprod Sci.

[15] Alexandre-Gouabau M.C., Courant F., Moyon T. (2013). Maternal and cord blood LC-HRMS metabolomics reveal alterations in energy and polyamine metabolism, and oxidative stress in very-low birth weight infants. J Proteome Res.

[16] Atzori L., Antonucci R., Barberini L. (2011). 1H NMR-based metabolomic analysis of urine from preterm and term neonates. Front Biosci (Elite Ed).

[17] Clark R.H., Kelleher A.S., Chace D.H., Spitzer A.R. (2014). Gestational age and age at sampling influence metabolic profiles in premature infants. Pediatrics.

[18] Ryckman K.K., Berberich S.L., Shchelochkov O.A., Cook D.E., Murray J.C. (2013). Clinical and environmental influences on metabolic biomarkers collected for newborn screening. Clin Biochem.

[19] Bennett M.J. (2014). Newborn screening for metabolic diseases: saving children's lives and improving outcomes. Clin Biochem.

[20] Clinical Laboratory Standards Institute (2013). Blood Collection on Filter Paper for Newborn Screening Programs; Approved Standard-Sixth Edition. CLSI document NBS01-A6.

[21] Liu L., Johnson H.L., Cousens S. (2012). Global, regional, and national causes of child mortality: an updated systematic analysis for 2010 with time trends since 2000. Lancet.

[22] Callaghan W.M., MacDorman M.F., Rasmussen S.A., Qin C., Lackritz E.M. (2006). The contribution of preterm birth to infant mortality rates in the United States. Pediatrics.

[23] Ezzati M., Lopez A.D., Rodgers A., Vander Hoorn S., Murray C.J. (2002). Comparative Risk Assessment Collaborating Group. Selected major risk factors and global and regional burden of disease. Lancet.

[24] Mitanchez D. (2007). Glucose regulation in preterm newborn infants. Hormone Res.

[25] Wu G., Imhoff-Kunsch B., Girard A.W. (2012). Biological mechanisms for nutritional regulation of maternal health and fetal development. Paediatr Perinat Epidemiol.

[26] Moltu S.J., Sachse D., Blakstad E.W. (2014). Urinary metabolite profiles in premature infants show early postnatal metabolic adaptation and maturation. Nutrients.

[27] Kc A., Wrammert J., Nelin V., Ewald U., Clark R., Malqvist M. (2015). Level of mortality risk for babies born preterm or with a small weight for gestation in a tertiary hospital of Nepal. BMC Public Health.

[28] Vrachnis N., Botsis D., Iliodromiti Z. (2006). The fetus that is small for gestational age. Ann N Y Acad Sci.

[29] Tyson J.E., Kennedy K., Broyles S., Rosenfeld C.R. (1995). The small for gestational age infant: accelerated or delayed pulmonary maturation? Increased or decreased survival?. Pediatrics.

[30] Giapros V., Drougia A., Krallis N., Theocharis P., Andronikou S. (2012). Morbidity and mortality patterns in small-for-gestational age infants born preterm. J Matern Fetal Neonatal Med.

[31] McIntire D.D., Bloom S.L., Casey B.M., Leveno K.J. (1999). Birth weight in relation to morbidity and mortality among newborn infants. N Engl J Med.

[32] Martin J.A., Osterman M.J., Kirmeyer S.E., Gregory E.C. (2015). Measuring gestational age in vital statistics data: transitioning to the obstetric estimate. Natl Vital Stat Rep.

[33] Therrell B.L., Padilla C.D., Loeber J.G. (2015). Current status of newborn screening worldwide: 2015. Semin Perinatol.

[34] Ryckman K.K., Dagle J.M., Shchelochkov O.A. (2013). Association of amino acids with common complications of prematurity. Pediatr Res.

[35] Ryckman K.K., Spracklen C.N., Dagle J.M., Murray J.C. (2014). Maternal factors and complications of preterm birth associated with neonatal thyroid stimulating hormone. J Pediatr Endocrinol Metab.

[36] Kwan A., Puck J.M. (2015). History and current status of newborn screening for severe combined immunodeficiency. Semin Perinatol.

